# Comparison of percutaneous vs oral infection of hamsters with the hookworm *Ancylostoma ceylanicum*: Parasite development, pathology and primary immune response

**DOI:** 10.1371/journal.pntd.0010098

**Published:** 2022-01-05

**Authors:** Richard D. Bungiro, Lisa M. Harrison, Blaise Dondji, Michael Cappello

**Affiliations:** 1 Department of Pediatrics, Yale University School of Medicine, New Haven, Connecticut, United States of America; 2 Laboratory of Cellular Immunology and Parasitology, Department of Biological Sciences, Central Washington University, Ellensburg, Washington, United States of America; Istituto Superiore di Sanità, ITALY

## Abstract

**Background:**

Hundreds of millions of people in poor countries continue to suffer from disease caused by bloodfeeding hookworms. While mice and rats are not reliably permissive hosts for any human hookworm species, adult Golden Syrian hamsters are fully permissive for the human and animal pathogen *Ancylostoma ceylanicum*. Similar to humans, hamsters may be infected with *A*. *ceylanicum* third-stage larvae orally or percutaneously. Oral infection typically leads to consistent worm yields in hamsters but may not accurately reflect the clinical and immunological manifestations of human infection resulting from skin penetration.

**Methodology/Principal findings:**

In this study we compared host responses following percutaneous infection to those utilizing an established oral infection protocol. Infected hamsters exhibited a dose-dependent pathology, with 1000 percutaneous larvae (L3) causing anemia and adult worm recovery comparable to that of 50 orally administered L3. A delayed arrival and maturity of worms in the intestine was observed, as was variation in measured cellular immune responses. A long-term study found that the decline in blood hemoglobin was more gradual and did not reach levels as low, with the nadir of disease coming later in percutaneously infected hamsters. Both groups exhibited moderate growth delay, an effect that was more persistent in the percutaneously infected group. Fecal egg output also peaked later and at lower levels in the percutaneously infected animals. In contrast to orally infected hamsters, antibody titers to larval antigens continued to increase throughout the course of the experiment in the percutaneous group.

**Conclusions/Significance:**

These results demonstrate that the route of infection with *A*. *ceylanicum* impacts disease pathogenesis, as well as humoral and cellular immune responses in an experimental setting. These data further validate the utility of the Golden Syrian hamster as a model of both oral and percutaneous infection with human hookworms.

## Author summary

Hookworms are bloodfeeding intestinal parasites that represent an important cause of anemia and growth delay in children from Low and Middle Income Countries (LMICs). The study of hookworm is limited by the limited availability of animal models that accurately reproduce the clinical features of human infection. We report here a detailed description of hookworm infection in the hamster, with comparison of two routes of infection: oral vs percutaneous. The results demonstrate differences in the time course of infection and primary immune responses based on whether infection occurs orally or via skin penetration. These results build on the current understanding of hookworm pathogenesis and extend the utility of this important animal model of human disease.

## Introduction

Despite significant improvements in public health over the past century, hundreds of millions of persons worldwide continue to suffer from disease caused by hookworms [1–3]. These bloodfeeding nematodes are a major cause of iron deficiency anemia and rank among the foremost agents of global morbidity [1,2], with a particular burden on children [4]. Although moderately effective anthelminthic agents for hookworm have been available for many years, reinfection typically occurs quickly following treatment [5–7], and reduced effectiveness has been documented in human infections [8–12]. In humans, infection with hookworms typically occurs by one of two potential routes, either through ingestion (*Ancylostoma*) of infectious third stage larvae (L3) or via skin penetration (*Necator americanus* or *Ancylostoma*) following contact with soil or vegetation contaminated with L3 [13,14]. Studies in mice utilizing the dog hookworm *A*. *caninum* suggest that hydrolytic enzymes, including proteases and hyaluronidases, facilitate larval skin penetration [15–17] and *in vitro* assays have demonstrated that antibodies directed at proteins secreted by *A*. *caninum* L3 may inhibit this process [17–19]. Following invasion of small cutaneous blood vessels, the worms migrate through the heart and lungs, where they lodge in the small capillaries. After entering the alveolar space parasites ascend into the large airways and upon reaching the trachea are swallowed, undergoing two additional molts to the adult bloodfeeding stage.

While mice and rats are not permissive hosts for the major human-infecting species *N*. *americanus* and *A*. *duodenale*, immunocompetent adult Golden Syrian hamsters (HsdHan:Aura) are fully permissive hosts for *A*. *ceylanicum* [20–22], an emerging pathogen of humans in southeast Asia [23–26]. When orally infected with a sublethal dose of *A*. *ceylanicum* L3, weanling hamsters exhibit the hallmarks of human infection: delayed growth and anemia [27]. Furthermore, we have demonstrated by ELISA and Western immunoblot that outbred hamsters undergoing a primary *A*. *ceylanicum* infection acquire vigorous specific serum IgG responses to soluble adult hookworm somatic extracts and adult excretory-secretory (ES) products [27–30].

In the experiments described here, the *A*. *ceylanicum* hamster model was used to investigate hookworm pathogenesis and host responses induced by larval skin penetration. Percutaneous infection of hamsters leads to the establishment of patent infections in infected animals and through detailed observations, the time course of parasite development and associated host pathology, including immune responses are characterized in comparison to animals infected using the oral infection protocol.

## Materials and methods

### Ethics statement

All experiments described were reviewed and received prior approval (protocol #10312) from the Yale University Animal Care and Use Committee. Daily maintenance and care of experimental animals complied with the National Institutes of Health guidelines for the humane use of laboratory animals and were provided by the Yale Animal Care and Resource Center Staff.

### Parasites and hosts

The *Ancylostoma ceylanicum* life cycle was maintained in male weanling (21 days old) hamsters of the outbred HsdHan:AURA strain (Envigo, Indianapolis, IN) through oral infection with 150–200 third-stage larvae (L3) freshly isolated from coprocultures [27]. For percutaneous infections, animals were anesthetized, and a small (2 cm square) patch of abdominal skin shaved with veterinary clippers. Larvae were suspended in a small volume of buffer and applied to the gauze pad of a Band-Aid Water Block bandage (Johnson & Johnson, New Brunswick, NJ). The L3-containing bandage was then applied to the exposed skin, secured with a layer of surgical tape wrapped around the animal’s body and left in place for 24 hours.

Three separate experiments were conducted to evaluate the course of disease and immune responses in percutaneously infected animals. In Experiment #1, the pathogenesis of percutaneous infection in hamsters (n = 3 per group) was examined in animals infected with various doses (50, 200, 1000, or 5000 L3). A separate group of animals was orally infected with 50 L3 to serve as infection controls. Blood hemoglobin levels were measured in all animals for 21 days. At day 22 post-infection (PI), all hamsters were sacrificed and adult worms in the small intestine were counted. Feces were collected from each animal following sacrifice to determine individual egg counts.

In the second experiment (Experiment #2), the time course of acute infection in animals infected percutaneously and orally was compared. Pathology and immune responses were measured in orally infected animals (n = 9; 100L3) and compared to observations of hamsters infected through the skin with 2000 L3 (n = 9) as described above. At days 10, 20 and 30 PI, 3 hamsters from each group were euthanized and adult hookworms removed, photographed, and measured. Spleens and mesenteric lymph nodes were removed, weighed and processed for FACS analysis as described below [31].

In Experiment #3, long-term disease progression and recovery were characterized by infecting separate groups of animals (n = 6) orally (50 oral L3) or via exposure through the skin (1000 percutaneous L3). Weight and blood hemoglobin levels were recorded for 100 days PI and compared to values for control animals that were uninfected. Pooled fecal samples were collected from infected animals for enumeration of parasite egg output.

### Measurement of fecal egg counts

Feces were collected from the large intestines of animals following sacrifice or from live animals temporarily housed on wire grating [32]. Eggs were counted in individual or pooled samples by the method of Gordon and Whitlock [33]. Briefly, fecal samples were mixed and 1 g suspended in 10 ml saturated NaCl, vortexed for 1 min and filtered through two layers of gauze. Eggs contained in the top layers of filtrate were counted in each chamber of a McMaster slide (Hausser Scientific, Horsham, PA) and viewed using light microscopy. Three separate preparations were analyzed to determine mean eggs per gram (epg) of feces for each infected animal or group.

### Flow cytometry

Single-cell preparations of spleens and mesenteric lymph nodes were prepared from tissues recovered from sacrificed animals in experiment 2 [31]. Approximately 10^6^ cells were surface stained with fluorescein isothiocyanate-labeled goat anti-Syrian hamster immunoglobulin G (FITC-IgG) and phycoerythrin-labeled anti-mouse CD4 [PE-L3T4; eBiosciences (San Diego, CA)] previously shown to interact with hamster cells [31,34]. Cell surface determinant data were acquired for 10^5^ cells per sample, using a FACSCalibur flow cytometer (BD Biosciences, San Jose, CA). Lymphocytes were selected by forward scatter gating on size, and data were analyzed using the FlowJo software program (Treestar, Ashland, OR).

### Parasite antigens

Soluble excretory-secretory products (ES) were obtained from adult hookworms collected from the intestines of life cycle animals. Orally infected animals were sacrificed 21 days PI and adult worms removed, rinsed with sterile PBS and incubated at 37°C in sterile PBS (10 worms per milliliter) as described [27,35]. Several preparations of frozen ES were thawed, pooled and filtered (0.45 micron) prior to concentration using a centrifugal concentrator with a 5-kDa molecular weight cut-off (Millipore Corp., Bedford, MA). Aliquots of concentrated ES were stored at -80°C until use.

Soluble hookworm larval extracts (LEX) were prepared from approximately 25,000 *A*. *ceylanicum* L3, which were suspended in 50 mM Tris HCl (pH 7.5) before flash freezing in dry ice ethanol. Incubation at 37°C for 5 minutes following freezing was performed twice. Insoluble material was pelleted by centrifugation (15,000x g) for 15 minutes at 4°C. The soluble supernatants were combined and aliquots stored frozen -80°C until use.

Recombinant *Ancylostoma ceylanicum* excretory-secretory protein 2 (rAceES-2) expression was induced in transformed *E*.*coli* (BL21 [DE3] strain) and soluble protein purified from cell pellets using sonication and chelation chromatography [28]. Elution fractions containing recombinant protein were dialyzed into PBS prior to -80°C storage.

### Enzyme-linked immunosorbent assays (ELISAs)

Serum samples were obtained from animals throughout the course of the Experiment #3 for the evaluation of systemic parasite-specific antibodies. Previously frozen adult worm ES was diluted to 2 μg/ml in sterile PBS and 100 microliters were added to individual wells of a 96 well Immulon-2HB microtiter plate (Dynex, Chantilly, VA) overnight at 4°C. The plates were washed 4 × with PBS-T (PBS and 0.05% Tween-20) and blocked for 1h at RT with 1% milk in PBS. Blocked plates were washed and then incubated for 3h at RT with serum serially diluted in 1% milk/PBS-T to a final volume of 100 μL/well. Plates were washed 6 × and incubated for 2h at RT with HRP-conjugated goat anti-hamster IgG (1:1000) in 1% milk/PBS-T. Bound HRP was visualized in washed plates by the addition of 100 μ L/well substrate solution (1 mg/mL ABTS [2,2′-azinobis (3-thylbenzthiazoline- 6-sulphonic acid); Sigma] in 0·1 M citrate buffer, pH 5·0, 0·03% H2O2). After 30 minutes incubation, the Abs_405_ was recorded using a SpectraMax 190 microplate reader (Molecular Devices, Sunnyvale, CA). All values were normalized to a positive standard which was included in each assay to control for day-to-day variation.

### Statistics

Numerical data is presented in the figures as means of individual or pooled samples as indicated ± SD. Statistical analysis was performed using the GraphPad Prism v6 software package (GraphPad Software, San Diego, CA) with specific tests indicated in the figure legends. In all cases P-values of < 0.05 were considered significant.

## Results

### Experiment #1: dose response

**Fig 1:**
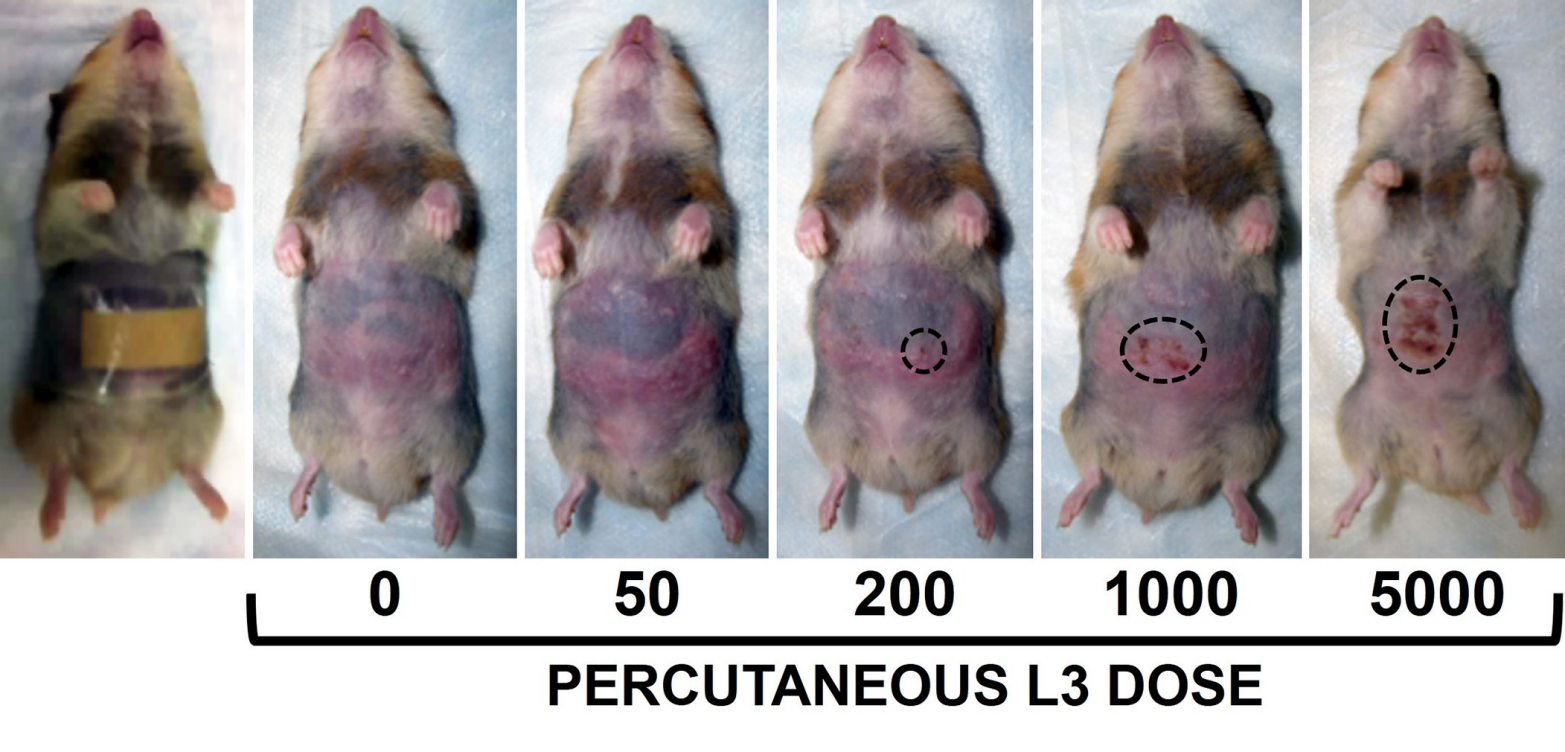
Representative photographs of hamsters following percutaneous infection, Experiment 1. Left panel shows the application of larvae to the abdominal area using an adhesive bandage as described in the Materials and Methods. Remaining panels show animals two days after infection with the indicated number of hookworm larvae. Dotted lines indicate extent of skin reaction to larval penetration.

**Fig 2:**
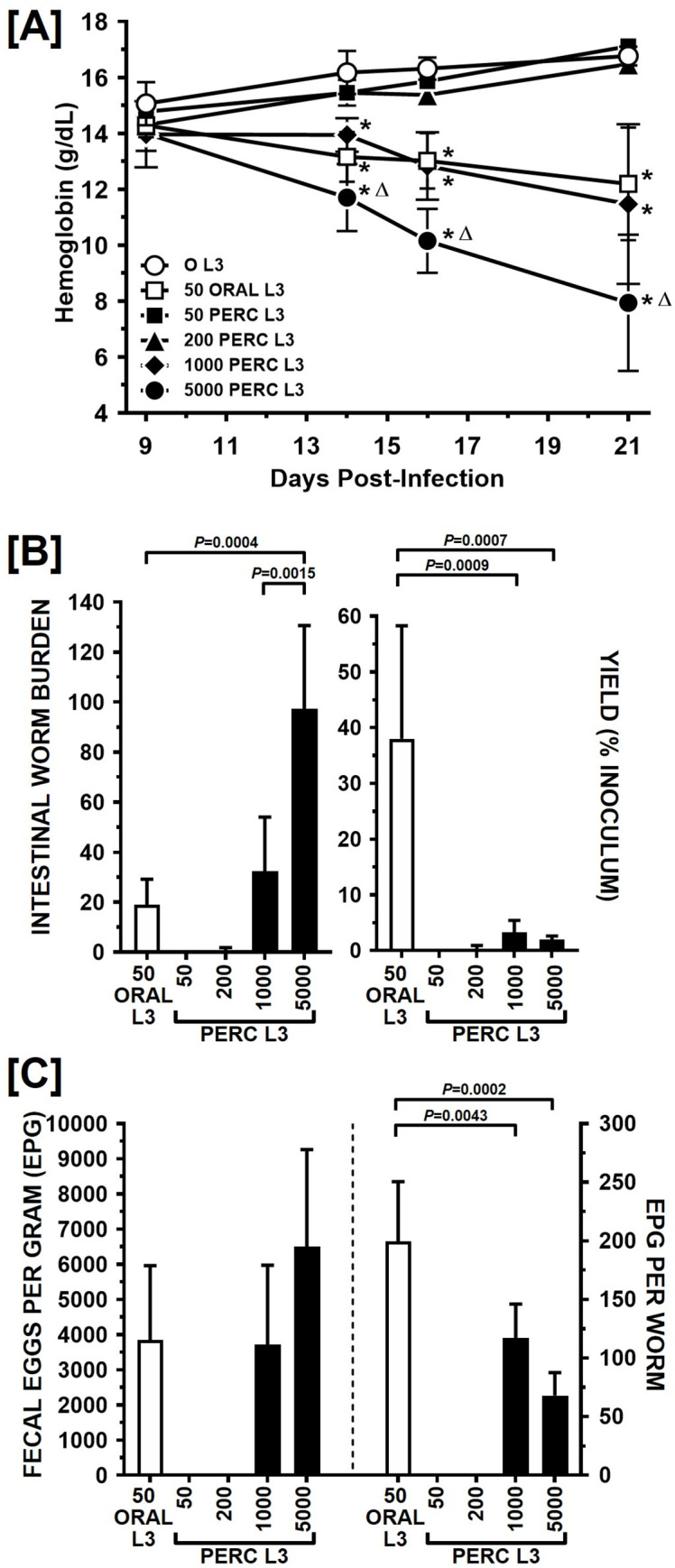
Pathology, worm burden and fecal egg output following percutaneous infection (Experiment 1). Hamsters (n = 3 each group) were infected with the indicated number of *A*. *ceylanicum* L3 by oral or percutaneous (PERC) route. Mean blood hemoglobin levels +/- Standard Deviation (SD) are presented in [A], intestinal worm burdens at day 22 PI are shown in [B] as absolute numbers (left) or yield expressed as percent of inoculum (right), and mean fecal egg output in [C] as calculated per gram of feces (EPG) recovered from the large intestines of individual animals (left) or EPG per recovered worm (right). Symbols in [A] indicate level of statistical significance by Fisher’s Least Significant Difference post-test following a two-way ANOVA: for the comparison to uninfected (0 L3) animals * indicates *P*< 0.05. For the comparison of 1000 L3 percutaneous and 5000 L3 percutaneous groups, △ indicates *P*< 0.05. In [B] and [C] brackets indicate statistically significant comparisons by one-way ANOVA, with P values given above the brackets.

We first conducted an experiment in which weanling hamsters were percutaneously infected with a range of larval doses, with orally infected and uninfected hamsters serving as controls. Percutaneous infection of weanling hamsters resulted in a dose-dependent acute skin reaction visible by 2 days after exposure to larvae (Fig 1). As the infection proceeded, hamsters percutaneously infected with the lowest doses, 50 and 200 L3, displayed no overt pathology, having blood hemoglobin levels statistically equivalent to those of uninfected control animals (Fig 2A). This contrasted with hamsters receiving higher percutaneous doses (1000 or 5000 L3), which exhibited reductions in hemoglobin levels compared to uninfected controls (Fig 2A). Animals percutaneously infected with 1000 L3 displayed moderate reductions in hemoglobin (2.5 g/dl decrease in mean levels by day 21 post infection as compared to day 9 levels, representing a 17.9% decline), similar to those receiving 50 L3 orally (2.1 g/dL decrease, or -14.6%). Severe pathology, however, was observed in animals receiving 5000 L3 percutaneously, with steep declines in hemoglobin compared to the uninfected control group and all other infected groups (Fig 2A). Blood hemoglobin levels in the 5000 percutaneously infected hamster ultimately reached the lowest levels measured in any group (6.1 g/dL drop from day 9 to 21, a 43.3% decrease).

At the time of sacrifice, the mean +/- SD adult intestinal worm burden (Fig 2B, left) at 22 days PI was 19.0 +/- 10.1 in the 50 oral L3 group, representing a 38.0% yield of the initial inoculum (Fig 2B, right) and consistent with previous findings [27,28,35,36]. By comparison, the mean worm burden in the 1000 L3 percutaneously infected group (32.3 +/- 21.7) was statistically equivalent to that of the 50 L3 oral animals, but the yield (3.2%) was more than 10X lower. The mean worm burden (97.3 +/- 33.3) in the 5000 L3 percutaneously infected group was considerably higher than either the 50 oral L3 or 1000 L3 percutaneous groups, although the overall yield of adult worms (1.9%) was equivalent that of the 1000 L3 percutaneous group. Consistent with the lack of observable pathology in the lowest dose percutaneous groups, no worms were recovered from any of the 50 L3 animals and only one of the 200 L3 animals was found to be infected, harboring two worms.

Consistent with the pathology and worm burden data, measurement of fecal eggs in individual animals demonstrated that the 50 oral L3 and 1000 percutaneous L3 groups had equivalent absolute counts (3855 +/- 2108 vs. 3717 +/- 2261 epg; Fig 2C left). Likewise, the highest egg counts were measured in the 5000 percutaneous L3 group (6500 +/- 2763 epg), although the difference was not statistically significant when compared to other groups. However, calculation of epg per recovered worm (Fig 2C right) revealed a higher ratio in the 50 oral L3 group (200 +/- 51) compared to the 1000 and 5000 percutaneous L3 groups (117 +/- 29 and 68 +/- 19, respectively). No eggs were observed in any fecal sample from 50 and 200 percutaneous L3 groups.

### Experiment 2: time course of primary infection

**Fig 3:**
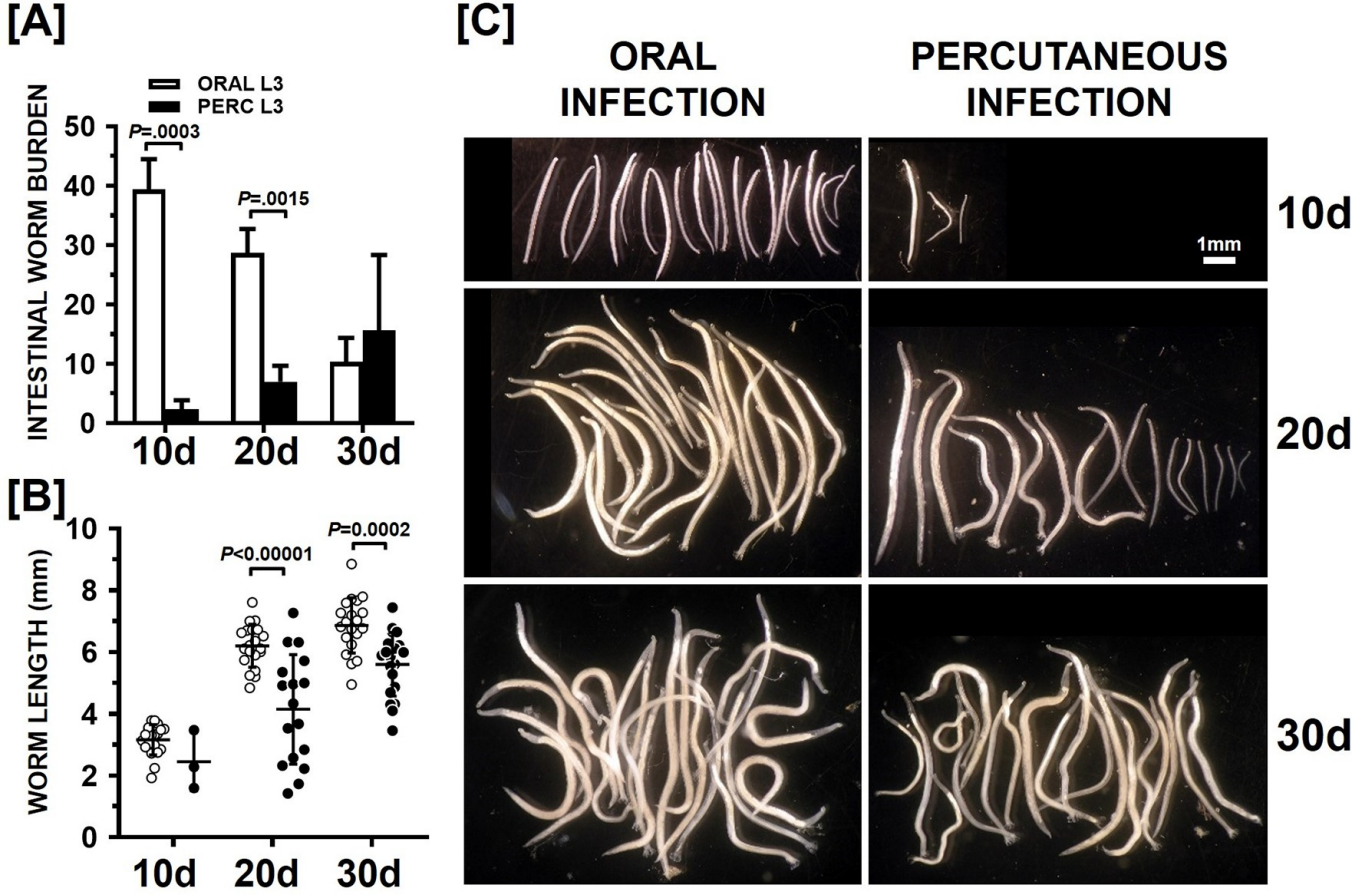
Intestinal worm burden and worm lengths following percutaneous infection (Experiment 2). Hamsters (n = 9 each group) were infected with 100 L3 by the oral route or 2000 L3 by the percutaneous (PERC) route. At days indicated, three animals from each group were sacrificed and intestinal worms recovered, counted and measured. Mean +/- SD intestinal worm burdens at each time point are shown in [A], worm length at each time point in [B] and representative photographs of worms recovered at each time point in [C]. In [A] and [B] brackets indicate statistically significant comparisons by Welch t test, with P values given above the brackets.

In the second set of experiments, parasite development and host responses were compared in animals infected orally with 100 L3 or 2000 L3 administered on the gauze bandage as described above. Animals were sacrificed on days 10, 20 and 30 post infection and the number of adult worms were counted and worm lengths measured. The spleens and mesenteric lymph nodes (MLN) from all animals were weighed and cellular profiles acquired. Adult worm yields were significantly higher on days 10 and 20 PI in the orally infected animals (p = 0.0003 and p = 0.0015; Fig 3A), with values declining at each time point. The lowest values were observed at 30 days PI. In contrast, the worm burdens in the animals percutaneously infected with 2000 L3 increased over the course of the experiment with highest values seen 30 days PI (Fig 3A).

The rise over time in the number of adult worms recovered from the small intestines in skin infected animals is likely due to larval migration and development within host tissues and the corresponding staggered maturity to bloodfeeding adults. This hypothesis is supported by the worm length data (Fig 3B and 3C) that shows while worms collected from the intestines of these animals were smaller at 20 and 30 days PI (Fig 3B), worm lengths continued to increase over the course of the experiment.

**Fig 4:**
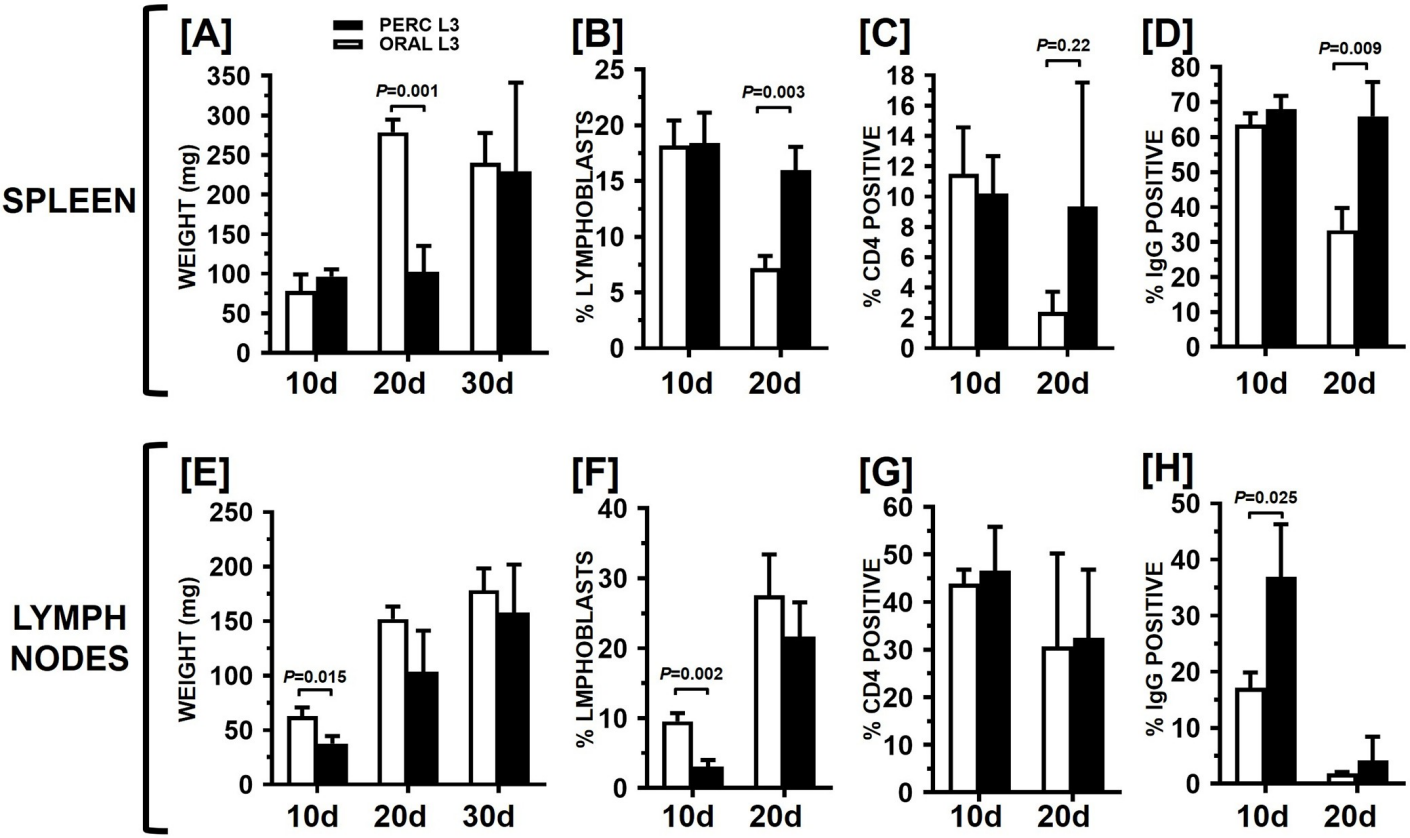
Organ weight and cellular data following percutaneous infection (Experiment 2). Hamsters (n = 9 each group) were infected with 100 L3 by the oral route or 2000 L3 by the percutaneous route. At days indicated, 3 animals from each group were sacrificed and organs recovered, weighed, and lymphocytes analyzed by flow cytometry. For the spleen, mean organ weights are shown in [A], percentage of blasting lymphocytes in [B], percentage of CD4-positive lymphocytes in [C] and percentage of IgG-positive lymphocytes in [D]; the corresponding data for mesenteric lymph nodes is shown in [E-H]. In all panels brackets indicate statistically significant comparisons by Welch t test, with P values given above the brackets. Bars represent Mean values +/- SD.

Spleens and MLNs from necropsied animals were recovered, weighed and lymphocytes prepared for analysis for surface IgG and CD4 expression. By 20 days PI, spleen weights from the orally infected hamsters had increased more than threefold over the day 10 PI values, and were more than double that of percutaneously infected animals (p = 0.001; Fig 4A). By 30 days PI, however, spleen weights in the percutaneously infected animals had more than doubled to become statistically equivalent to the orally infected animals. The mean weights of MLNs of both groups increased over the duration of the experiment, with a significant difference seen only at day 10 when the MLNs from orally infected animals were larger (p = 0.015; Fig 4E).

Cellular immune responses in the spleen and MLNs were measured at 10 and 20 days PI. The day 30 PI data could not be obtained due to a technical issue leading to loss of samples prior to FACS analysis. The percentage of lymphoblasts in the spleen (Fig 4B) was equivalent between the two infection groups at 10 days PI, but decreased to a significantly lower level in the orally infected group at 20 days PI (p = 0.003). Conversely, the percentage of blasts in the MLNs (Fig 4F) was greater in orally infected hamsters (p = 0.002) at 10 days PI, but in both groups had increased to equivalent levels by day 20 PI. The mean percentage of CD4+ lymphocytes in the spleen (Fig 4C) decreased almost fivefold in orally infected hamsters from 10 to 20 days PI and was lower than that of the percutaneously infected animals at 20 days PI; in the MLN the mean CD4+ percentage remained relatively stable and equivalent between the two groups (Fig 4G). The mean percentage of surface IgG+ lymphocytes in the spleen (Fig 4D) decreased by almost half in orally infected hamsters from 10 to 20 days PI and was lower than that of the percutaneously infected animals at day 20 PI (p = 0.009). Of note, the mean percentage of IgG+ lymphocytes in the MLNs of percutaneously infected hamsters (Fig 4H) was twofold higher than orally infected animals at day 10 PI (p = .025), but by 20 days PI the mean IgG+ percentage of both groups had declined almost tenfold and were statistically equivalent.

### Experiment 3: long term disease progression and recovery

**Fig 5:**
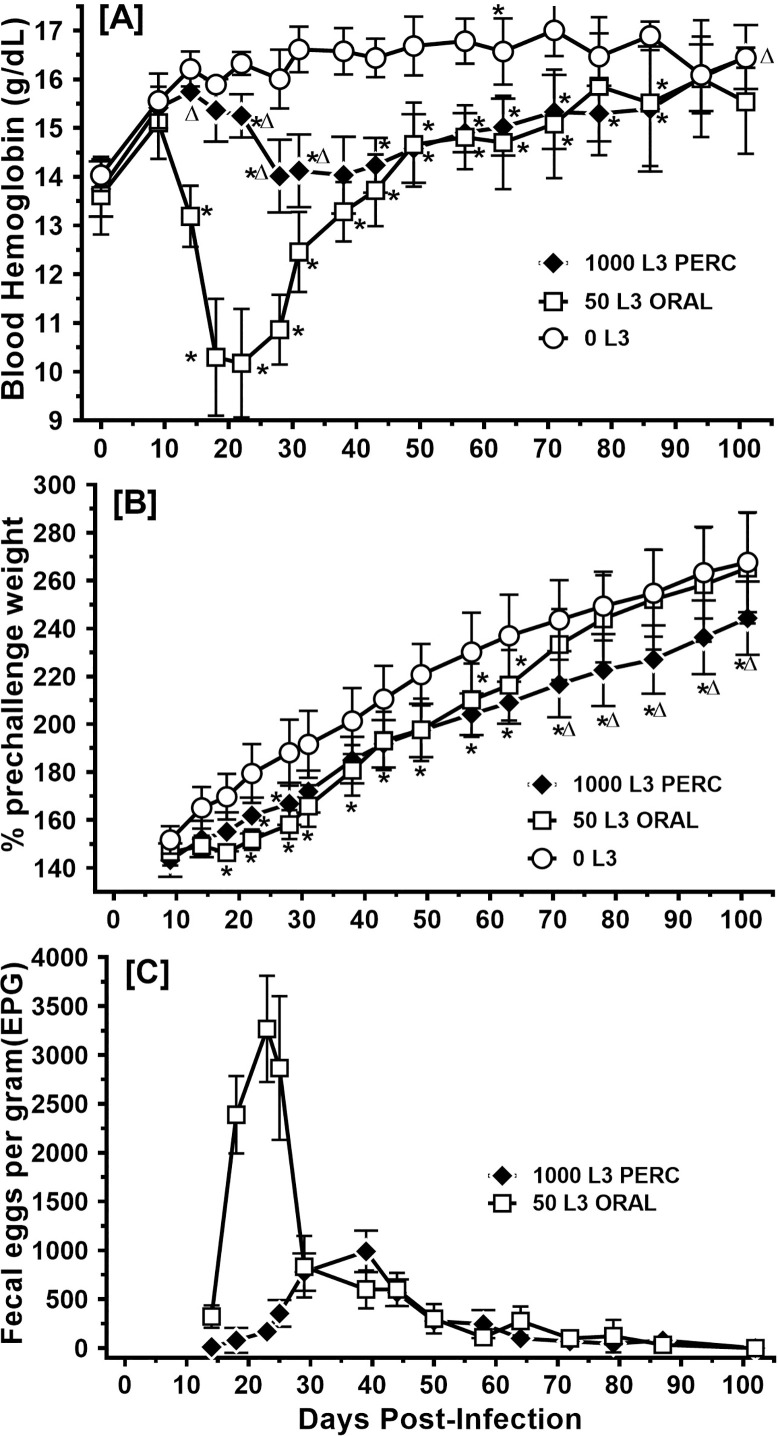
Pathology and fecal egg output following percutaneous infection (Experiment 3). Hamsters (n = 6 each group) were infected with the indicated number of L3 by oral or percutaneous route. Mean blood hemoglobin levels over time are presented in [A], weight change over time in [B] and pooled fecal egg output in [C]. Data points represent Mean +/- SD. Symbols in [A] and [B] indicate level of statistical significance by Fisher’s Least Significant Difference post-test following a two-way ANOVA: for the comparison to 0 L3 * indicates *P*< 0.05. For the comparison of 50 L3 oral and 1000 L3 percutaneous groups, △ indicates *P*< 0.05.

A long-term infection of hamsters comparing sublethal oral and percutaneous infections was used to evaluate progression of disease parameters (weight and blood hemoglobin) and fecal egg excretion, as well as the development of systemic antibody responses to parasite antigens. Animals receiving oral doses of infectious larvae exhibited a characteristic rapid decline in blood hemoglobin (Fig 5A) and growth delay (Fig 5B), reaching a nadir approximately 3 weeks PI, which also coincided with peak fecal egg excretion in this group (Fig 5C). Orally infected hamsters then showed signs of recovery as evidenced by improvements in both disease parameters and coincident decline of fecal egg output. Hemoglobin levels of percutaneously infected animals also declined, however compared to orally infected hamsters the decline was more gradual, less pronounced, and the nadir of disease (28 days PI) occurred later (Fig 5A). Like orally infected animals, percutaneously infected animals exhibited moderate growth delay, although this effect was more persistent in the percutaneously infected group (Fig 5B). Fecal egg output peaked later in the percutaneously infected animals and never exceeded 1000 epg; by contrast the levels in orally infected animals peaked above 3000 epg by day 23 PI (Fig 5C).

**Fig 6:**
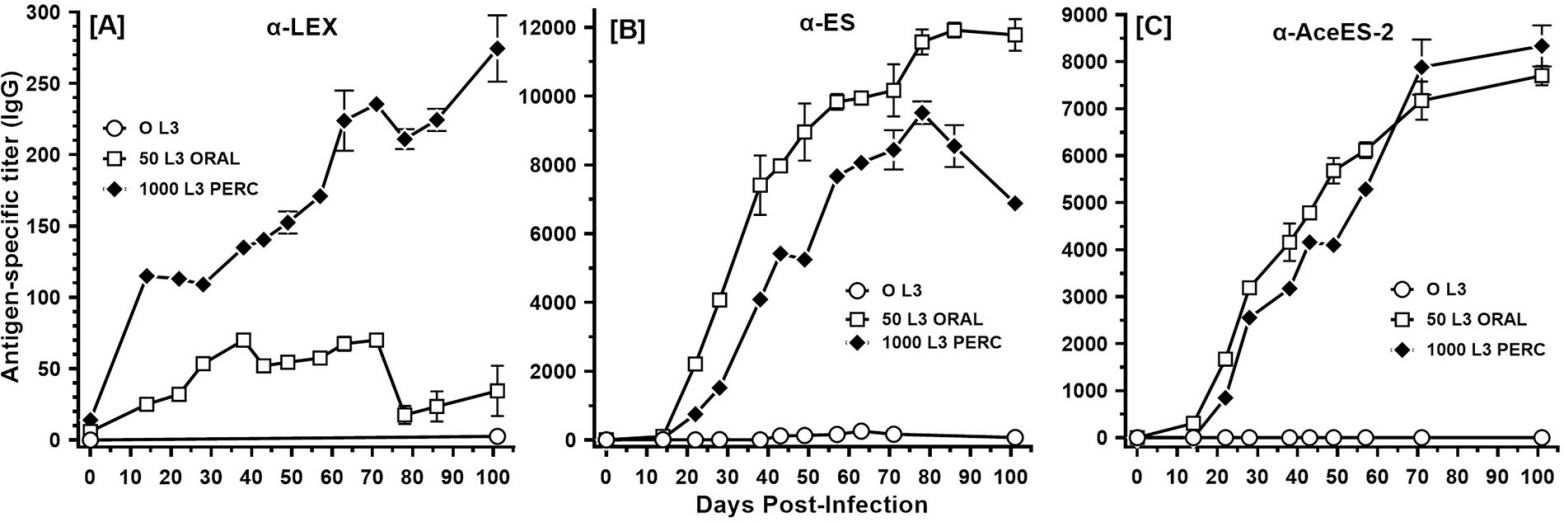
Hookworm-specific antibody responses following percutaneous infection (Experiment 3). Hamsters (n = 6 each group) were infected with the indicated number of L3 by oral or percutaneous route. Specific IgG titers against soluble larval extract (LEX) in pooled hamster sera are presented in [A], titers against adult worm excretory-secretory (ES) antigens are presented in [B], and rAceES-2-specific titers in [C]. Data points represent Mean +/- SD.

The onset and progression of systemic antibody titers to worm antigens from different life stages varied between exposure groups, with higher titers to larval antigens seen in percutaneously infected animals (Fig 6A). In those animals, titers to larval antigens continued to increase throughout the course of the experiment. In contrast, higher titers to adult ES antigens were present throughout the experiment in orally infected animals (Fig 6B). Antibody responses to the highly immunogenic ES antigen AceES-2 exhibited similar kinetics in both groups of infected animals (Fig 6C), suggesting that route of infection has little effect on the development of systemic IgG to this ES antigen.

## Discussion

Among the challenges facing parasitologists is access to reproducible animal models that accurately reflect the major clinical features of human disease. The study of hookworm highlights this difficulty, as the two most common human parasite species (*Necator americanus* and *Ancylostoma duodenale*) do not reliably complete their life cycle in laboratory mice or rats. There are reports in the literature describing the use of rabbit kits [37], puppies [38], neonate hamsters [39], and/or the use of immunosuppressive agents [40] for the characterization of infections with human hookworms. While such studies have advanced the field of parasitology, pathological and immune response data collected from these systems may be challenging to generalize to human infection and disease.

Although primarily a zoonotic pathogen of cats and dogs, *Ancylostoma ceylanicum* is the second most common hookworm infecting people in Southeast Asia [26,41,42]. Studies using the hamster model suggest that oral infections with *Ancylostoma ceylanicum* [26,41] closely approximate some observations made in human infections [27,31,32,43], which has added to our understanding of hookworm pathogenesis and the host-parasite interaction. Establishment of patent *A*. *ceylanicum* infections in outbred Golden Syrian hamsters has made possible detailed characterizations of hookworm disease pathogenesis [27], evaluation of therapeutic interventions [36,44–47], vaccine development [27,48–54], and characterization of innate/acquired immune responses [31,34,35,45].

Studies in the hamster model of *A*. *ceylanicum* that have utilized the oral infection route confirm that it is highly reproducible and has a relatively short pre-patent period (approximately 20 days) [27,55]. However, since *A*. *ceylanicum* larvae administered by oral gavage do not undergo the tissue migration that follows percutaneous infection [56], the model has important limitations for understanding certain aspects of pathogenesis, in particular systemic responses to larval invasion. The results presented here provide a more detailed analysis of the time course, inoculum-dependent pathology, and the immune response to primary percutaneous infection of Golden Syrian hamsters with *A*. *ceylanicum*. In the current set of experiments, we demonstrate dose-dependent pathology and worm burdens in percutaneously infected hamsters (Fig 2). As seen in experimental human infections [57,58], low intensity infections did not result in anemia or significant growth delay. Following percutaneous exposure to higher doses of hookworm larvae, we observed an expected onset of disease (anemia) correlating with the arrival and maturity of adult worms in the intestine (Fig 3) following larval tissue migration. From the parasitology perspective, these data demonstrate that the oral route is substantially more efficient than the percutaneous route, with higher yields and intensity of infection following oral dosing at 50 L3 comparable to an inoculum of 1000 L3 administered via the skin. One possible explanation for the difference is that tissue migration requires the parasite to evade multiple host defenses, including the skin and respiratory systems, both of which contain potent innate immune effectors. By contrast, when administered by oral gavage, the L3 undergo successive molts to the adult stage exclusively in the gastrointestinal tract [56], which may present fewer obstacles to further parasite development and survival of the adult worms. Although it’s possible that long term (>20 years) passage of this laboratory adapted strain of *A*. *ceylanicum* via oral infection of hamsters has resulted in a loss of infectious capacity via percutaneous exposure, our results are comparable to findings of Garside and Behnke [56], who also demonstrated substantially reduced yield of adult worms following percutaneous infection compared to oral gavage.

The staggered worm maturity and associated pathology observed following percutaneous infection were associated with impaired cellular immune responses (Fig 4), similar to prior investigations in the oral infection model [30,31,50]. Those studies demonstrated that oral infection with *A*. *ceylanicum* blunts the cellular and humoral immune responses as observed in experimental human infections with *N*. *americanus* [59,60]. In humans, reinfection with hookworms also leads to reduced cellular immune responses and faster parasite establishment compared to primary infections [57]. This may account for the continued accrual of adult worms and the establishment of chronic infections in humans living in endemic areas. Interestingly, however, the immune cell profiles differed between oral and percutaneous infection models. For example, although the spleens of orally infected animals were significantly larger than those from percutaneously infected hamsters, the latter group exhibited much higher percentages of spleen lymphoblasts, CD4+ T cells and surface IgG+ B cells. For comparison, previous studies have demonstrated that oral infection with *A*. *ceylanicum* is associated with a reduction in CD4+ splenocytes and CD4+ MLNs, as well as reduced numbers of IgG+ B cells in the spleen, compared to uninfected controls at day 20 post-infection [31]. The results reported here are perhaps explained by the observation that oral infection is associated with a more synchronous and earlier exposure to adult worm antigens in the gut, while percutaneous infection is associated with a gradual accumulation of adult worms that takes place over a more protracted period of time. In addition, the data suggest that the predominant mechanism of hookworm associated immune suppression, at least in this model, most likely involves adult worms in the intestine rather than tissue migrating L3. The delayed and asynchronous establishment of adult worms in the intestine can also be surmised from the data in Fig 5, which shows distinct patterns of anemia, growth delay and fecal egg excretion between the two routes of infection. The two profiles are consistent with an earlier and more intense peak of adult worm bloodfeeding and egg excretion in the orally infected animals, similar to prior results in this model. By contrast, the drop in blood hemoglobin in the percutaneously infected animals is less pronounced, as is the pattern of fecal egg excretion. Of note, however, percutaneous infection appears to be associated with a more sustained period of growth delay compared to the oral infection route.

Not unexpectedly, higher anti-larval stage antibody titers were noted in percutaneously infected animals compared to those animals infected orally (Fig 6). These higher titers likely resulted from increased antigen exposure and accompanying processing by immune effector cells following the attrition and degradation of larvae in the course of tissue migration. Of note, however, antibody titers to L3 protein extracts continued to rise across the 100 day experiment. This result suggests that there is an ongoing source of immune stimulation, perhaps due to a reservoir of degraded or tissue arrested L3. Observations of infected animals exposed to trickle oral infections reveal similar patterns of higher larval antibody titers corresponding to continued susceptibility to infection [61]. Also of note is the late decline in IgG antibodies directed at adult worm ES in the percutaneous infection group, a result that could be explained by reduced numbers of adult worms compared to the oral infection group. Despite these differences, we observed nearly identical IgG responses in both groups to *A*. *ceylanicum* ES-protein 2 (AceES-2) a highly antigenic, netrin-like protein secreted by adult hookworms in the hamster host [28,62,63]. This result suggests that AceES-2 is a persistent stimulus for antibody production in the hamster, regardless of the route of exposure. How this novel parasite secretory protein mediates pathogenesis and/or acquired immunity to hookworm *in vivo* remains to be determined.

We propose that infection of hamsters with hookworm larvae by the percutaneous route provides an alternative and biologically relevant experimental model of human hookworm disease, one that provides insight into features of infection resulting from this route of exposure. Unlike the hookworm *Necator americanus*, *A*. *duodenale* and *A*. *ceylanicum* are also infectious orally, although it is challenging to define the ratio of oral vs percutaneous infections occurring in human populations. Further characterizing both routes of infection and defining host responses that are unique to each should lead to a better understanding of hookworm immunoepidemiology and transmission dynamics in endemic populations. The continued characterization of the Syrian hamster model to include studies such as these, coupled with the expanded availability of reagents and methods for this rodent host species [64,65] will also provide a useful foundation for future work focused on developing effective therapeutic drugs, preventive vaccines and diagnostics.

## Supporting information

S1 Data Primary data from Experiment 1 (sheets 1–5), Experiment 2 (sheets 6–15), and Experiment 3 (sheets 16–21). (XLSX)
